# Severity and outcomes of Omicron variant of SARS-CoV-2 compared to Delta variant and severity of Omicron sublineages: a systematic review and metanalysis

**DOI:** 10.1136/bmjgh-2023-012328

**Published:** 2023-07-07

**Authors:** Pryanka Relan, Nkengafac Villyen Motaze, Kavita Kothari, Lisa Askie, Olivier Le Polain de Waroux, Maria D Van Kerkhove, Janet Diaz, Bharath Kumar Tirupakuzhi Vijayaraghavan

**Affiliations:** 1 COVID-19 Clinical Management, World Health Emergencies Programme, World Health Organization, Geneva, Switzerland; 2 Health Emergencies Programme, WHO, Geneva, Switzerland; 3 Medicine Usage in South Africa, School of Pharmacy, Faculty of Health Sciences, North-West University, Potchefstroom, South Africa; 4 Library and Digital Information Networks, World Health Organization, Kobe, Japan; 5 Methods and Standards Unit, Science Division, World Health Organization, Geneva, Switzerland; 6 Acute Response Coordination Department, World Health Organization, Geneva, Switzerland; 7 COVID-19 Health Operations, World Health Emergencies Programme, World Health Organization, Geneva, Switzerland; 8 Critical Care, Apollo Hospitals, Chennai, Tamil Nadu, India

**Keywords:** COVID-19, SARS-CoV-2, Critical Illness

## Abstract

**Objectives:**

To compare severity and clinical outcomes from Omicron as compared with the Delta variant and to compare outcomes between Omicron sublineages.

**Methods:**

We searched the WHO COVID-19 Research database for studies that compared clinical outcomes for patients with Omicron variant and the Delta variant, and separately Omicron sublineages BA.1 and BA.2. A random-effects meta-analysis was used to pool estimates of relative risk (RR) between variants and sublineages. Heterogeneity between studies was assessed using the I^2^ index. Risk of bias was assessed using the tool developed by the Clinical Advances through Research and Information Translation team.

**Results:**

Our search identified 1494 studies and 42 met the inclusion criteria. Eleven studies were published as preprints. Of the 42 studies, 29 adjusted for vaccination status; 12 had no adjustment; and for 1, the adjustment was unclear. Three of the included studies compared the sublineages of Omicron BA.1 versus BA.2. As compared with Delta, individuals infected with Omicron had 61% lower risk of death (RR 0.39, 95% CI 0.33 to 0.46) and 56% lower risk of hospitalisation (RR 0.44, 95% CI 0.34 to 0.56). Omicron was similarly associated with lower risk of intensive care unit (ICU) admission, oxygen therapy, and non-invasive and invasive ventilation. The pooled risk ratio for the outcome of hospitalisation when comparing sublineages BA.1 versus BA.2 was 0.55 (95% 0.23 to 1.30).

**Discussion:**

Omicron variant was associated with lower risk of hospitalisation, ICU admission, oxygen therapy, ventilation and death as compared with Delta. There was no difference in the risk of hospitalisation between Omicron sublineages BA.1 and BA.2.

**PROSPERO registration number:**

CRD42022310880.

**WHAT IS ALREADY KNOWN ON THIS TOPIC**

- While Omicron, currently the dominant variant of SARS-CoV-2, is known to have increased transmissibility and infectivity, true clinical severity remains uncertain.

**WHAT THIS STUDY ADDS**

- Our systematic review evaluates clinical severity and outcomes for individuals infected with Omicron compared with Delta as well as sublineages of Omicron (BA.1 and BA.2). Our results suggest that Omicron is associated with 61% lower risk of death and 56% lower risk of hospitalization. There were no differences in severity or clinical outcomes between Omicron sublineages.

**HOW THIS STUDY MIGHT AFFECT RESEARCH, PRACTICE OR POLICY**

- With waning immunity, changes in testing policies and procedures, cocirculation of numerous sublineages and variations in clinical management strategies, assessing severity for each new emerging variant is essential in order to optimise clinical care. Our findings thus provide important information for key stakeholders.

## Background

Globally, over 6.9 million deaths from COVID-19 have been reported to the WHO as of 24 May 2023.^1^ In the 3.5 years since the first reports of SARS-CoV-2, the virus has continuously evolved, and multiple variants of concern have emerged.^2^ While the emergence of future variants is a known and expected phenomenon, not all mutations confer a fitness advantage to the virus. Mutations that enhance pathogenicity, infectivity, transmissibility and/or antigenicity may confer important survival advantages, leading to newer and potentially deadlier waves of the pandemic.^3^ In parallel, it is important to recognise the changing host response as the pandemic continues, resulting in increasing population immunity due to prior infection(s) and the increasing availability of vaccines, as well as effective treatment options.^4–6^

In late November 2021, researchers from South Africa reported the emergence of a newer variant (B.1.1.529) to WHO after observing a rapid increase in cases from Gauteng province with the unique finding of S-gene target failure on PCR testing.^7^ The WHO Technical Advisory Group on SARS-CoV-2 Virus Evolution recommended classification as variant of concern (VOC) based on its transmissibility and immune escape properties. WHO named this VOC Omicron on 26 November 2021.^8^

Omicron rapidly replaced Delta to become the dominant circulating variant globally.^2^ While reports from several countries^9–11^ suggested that patients infected with Omicron were experiencing symptoms that were less severe than Delta, much of this information was unadjusted for potential confounding factors such as prior immunity from infection or vaccination and availability of COVID-19-specific therapeutics. Using information available in the Global Clinical Platform for COVID-19, WHO published a report (number of patients included 34 442) suggesting lower severity with Omicron as compared with Delta.^12^ Similar to other published reports, the WHO analysis also suffered from challenges such as small patient numbers from a limited number of settings and limited information on vaccination status and prior infection.

Despite the relative lower severity, the intense circulation of Omicron has resulted in significant mortality, with 1 243 850 reported deaths in 2022 globally.^1^ For WHO member states, public health institutions and the public, understanding the clinical impact of emerging variants is vital to ensure an appropriate public health response, including adequate resource allocation and to update clinical management guidelines. In this context and given the public health implications, the WHO Steering Committee for COVID-19 Clinical Guidelines requested a comprehensive review of the literature to better understand the severity of Omicron compared with previously circulating VOCs. The aim of this review was to compare severity and clinical outcomes for individuals infected with the Omicron variant as compared with the Delta variant and additionally between BA.1 and BA.1 sublineages of Omicron.

## Methods

### Review questions, inclusion criteria and outcomes

The initial review question was focused on the severity of Omicron as compared with the Delta variant. However, during the literature appraisal process, the emergence of sublineages BA.1 and BA.2 of Omicron triggered the expansion of the review to include severity of these sublineages (prompting an updated literature search). Therefore, review questions of interest were (1) among patients infected with Omicron variant, what is the severity and clinical outcomes as compared with patients infected with the Delta variant? and (2) among patients infected with the BA.2 sublineage of Omicron, what is the severity and clinical outcomes as compared with BA.1 sublineage?

Eligible studies were those with primary data on humans of any age group focusing on clinical features and clinical outcomes among patients with Omicron variant and Delta variant. Outcomes of interest were admission to hospital, admission to intensive care unit (ICU), receipt of oxygen therapy (low flow and high flow), receipt of non-invasive ventilation, receipt of invasive ventilation, receipt of kidney replacement therapy or any other organ support, and death at longest follow-up as reported in each study. Editorials, commentaries, viewpoints, letters (correspondence) and abstracts with no full text available were excluded.

### Search strategy

The search was developed and run by a Health Information Specialist (KK). The WHO COVID-19 Research database, a specialised, comprehensive COVID-19 resource, maintained by the WHO Library, was searched.^13^ This database includes peer reviewed publications from all major scientific databases (including Embase, MEDLINE and Web of Science), preprint articles and other grey literature sources.^14^ The initial search was run on 26 January 2022 with no date filters, and an update was run on 16 May 2022. No restrictions on language were applied. More details on the search strategy can be found in the online supplemental material 1.

10.1136/bmjgh-2023-012328.supp1 Supplementary data

### Study selection

Titles/abstracts of all retrieved citations and full texts were reviewed independently and in duplicate by two authors (PR and BKTV). In case of disagreement, a third reviewer (NVM) was available. Consensus was achieved for inclusion of articles.

### Data extraction

A standardised data extraction form was developed and piloted jointly by BKTV and PR prior to data extraction. Data were extracted on the number of patients for the different comparisons (Omicron vs Delta and BA.1 vs BA.2), on severity and clinical outcomes. In case of disagreement, a third reviewer (VM) was available. Consensus was achieved for data extraction.

### Study quality assessment

Study quality was assessed using the risk of bias (RoB) in observational studies tool developed by the Clinical Advances through Research and Information Translation research team.^15^ The tool assesses RoB across eight domains (selection of exposed and non-exposed from the same population, confidence in the assessment of exposure, confidence that outcome was not present at the start of the study, whether the study adjusted for the key covariates of interest, confidence in the assessment of presence or absence of prognostic factors, confidence in the assessment of outcome, adequacy of follow-up and similarity of cointerventions). Quality was assessed independently and in duplicate by two authors (PR and BKTV). In addition, we also developed rules to arrive at an overall quality rating (see online supplemental figure 8).

### Data analysis

All analyses were undertaken in R (R: A Language and Environment for Statistical Computing, R Core Team, R Foundation for Statistical Computing, Vienna, Austria; https://www.R-project.org).

Categorical variables are reported as counts and percentages and continuous variables as mean and SD (or median and IQR). We summarise qualitatively and quantitatively the severity and outcomes for patients by variant and sublineage. Hospitalisation is reported as a proportion of the entire study cohort of patients with Omicron and Delta, whereas in-hospital outcomes such as ICU admission, receipt of oxygen therapy, etc, are reported as a proportion of the total number of patients hospitalised for each variant.

The restricted maximum likelihood method, the default method in the *metafor* package V.3.0 in R,^16^ was used for analysis. We performed a random-effects meta-analysis for each outcome by combining the effect estimates of studies where they were available. When studies did not report effect estimates, we calculated the relative risk (RR) from the numbers of participants in each comparison group. In cases where studies reported the HR or OR, we calculated the risk ratio using the methods suggested by Schor *et al*^17^ and Zhang and Yu,^18^ respectively. For all studies, we extracted and pooled the adjusted estimates where available.

Heterogeneity between studies was assessed using the Q statistic with a significance level of p<0.10, and the I^2^ index was used to assess the degree of heterogeneity between study estimates.^19^ RoB plots were created using a standard R package for visualising RoB assessments.^20^

A post hoc sensitivity analysis was undertaken by pooling the results for different outcomes by incorporating only those studies that adjusted for vaccination and only studies at low RoB. We specified a significance level of p<0.05 for overall effect estimate in the meta-analysis for the random-effects model.

### Study registration and deviations from protocol

The review was registered prior to any data extraction and analysis on the International Prospective Register of Systematic Reviews (CRD42022310880).^21^

Our original review question did not include the Omicron sublineage comparison and was added after the widespread emergence of BA.2. The sensitivity analysis of studies that adjusted for vaccination and studies at low RoB was also not part of the original analysis plan.

We conducted and reported this review in accordance with Meta-Analyses of Observational Studies in Epidemiology (MOOSE) and Preferred Reporting Items for Systematic Reviews and Meta- Analyses (PRISMA) guidelines.^22 23^ We used Covidence to conduct this systematic review and meta-analysis (Covidence Systematic Review Software, Veritas Health Innovation, Melbourne, Australia).

### Patient and public involvement

Given the nature of this research, we did not involve patients or the public in the design or conduct or reporting.

## Results

Our search identified 1494 records, and after excluding duplicates and records not relevant to the study question, 57 full-text reports were assessed for eligibility. The PRISMA flow diagram (online supplemental material 1) provides the detailed reasons for exclusions. From the 57 full-text reports, 43 were included in the review^9–11 24–63^ and analysis. One additional study^60^ was excluded as the data reported in the paper was unclear and the study authors did not respond to emails, leaving us with 42 studies. A total of 6 174 807 patients were included in this review.

Table 1 describes the characteristics of the included studies. Five of six WHO regions were represented (Africa, Americas, Eastern Mediterranean, Europe and South-East Asia), and most studies were from high-income or upper-middle-income settings: 12 from the USA,^9 25 26 29 30 32 36 40 44 53 54 57^ 7 from the UK,^27 31 37 42 46 50 59^ 7 from South Africa,^33 51 52 55 56 58 61^ 5 from France^24 28 34 38 62^ and two from Norway.^35 39^ There was one study each from Denmark,^47^ Germany,^41^ India,^45^ Indonesia,^49^ Italy,^63^ Portugal,^10^Turkey^46^ and Qatar.^43^ Of the included studies, 11 were published as preprints.^6–8 19 25 32 44 45 49 52 56^ Sixteen studies reported on adult patients and 3 on children, and 21 studies included a combination of adults and children. Of the 42 studies, 29 adjusted for vaccination status either in the study design or in the analysis stage; 12 had no adjustment; and 1 study was unclear. Three of the included studies compared the sublineages of Omicron (BA.1 vs BA.2).^61–63^

**Table 1:** Characteristics of included studies

	First author	Country	WHO region	World Bank income	Year of publication	Publication type	Method used to identify variants	Participants, total n
Omicron versus Delta								
1	Abdullah *et al*^55^	South Africa	African	Upper middle income	2021	Peer reviewed	Time period	4428
2	Adhikari *et al*^40^	USA	Americas	High income	2022	Peer reviewed	WGS+time period	1343
3	Vieillard-Baron *et al*^24^	France	European	High income	2022	Preprint	Mutations in spike protein of SARS-CoV-2	3761
4	Auvigne *et al*^28^	France	European	High income	2022	Peer reviewed	RT-qPCR with mutation screening multiplex	184 364
5	Bager *et al*^47^	Denmark	European	High income	2022	Peer reviewed	RT-PCR using a specific Omicron marker	188 980
6	Bal *et al*^38^	France	European	High income	2022	Preprint+peer review	WGS	6223
7	Birol Ilter *et al*^46^	Turkey and UK	European	Upper middle income/high income	2022	Peer reviewed	Time period	231
8	Bouzid *et al*^34^	France	European	High income	2022	Peer reviewed	WGS+SGTF+detection of mutation	1716
9	Butt *et al*^43^	Qatar	Eastern Mediterraean	High income	2022	Peer reviewed	WGS+time period	1970
10	Ulloa *et al*^11^	Canada	Americas	High income	2021	Preprint	WGS+SGTF	25 802
11	Christensen *et al*^53^	USA	Americas	High income	2022	Preprint+peer review	WGS+SGTF	20 196
12	Fall *et al*^36^	USA	Americas	High income	2022	Preprint+peer review	WGS	2027
13	Goga *et al*^51^	South Africa	African	Upper middle income	2021	Preprint+peer review	Time period	39 929
14	Grint *et al*^50^	UK	European	High income	2022	Preprint	SGTF	330 380
15	Gunadi *et al*^49^	Indonesia	Southeast Asian	Lower middle income	2022	Preprint	WGS	352
16	Hussey *et al*^58^	South Africa	African	Upper middle income	2022	Preprint+peer review	RdRp target delay to detect Omicron	1636
17	Jassat *et al*^33^	South Africa	African	Upper middle income	2022	Preprint+peer review	Time period	1 935 877
18	Krutikov *et al*^59^	UK	European	High income	2022	Peer reviewed	WGS+SGTF+time periods with a subanalysis of confirmed	545
19	Lauring *et al*^29^	USA	Americas	High income	2022	Preprint+peer review	WGS+time period	1338
20	Leiner *et al*^41^	Germany	European	High income	2022	Preprint+peer review	Time period	3403
21	Lewnard *et al*^9^	USA	Americas	High income	2022	Preprint	SGTF	69 579
22	Davies *et al*^56^	South Africa	African	Upper middle income	2022	Preprint+peer review	Time period	9547
23	Menni *et al*^42^	UK	European	High income	2022	Peer reviewed	Time period	9980
24	Modes *et al*^26^	USA	Americas	High income	2022	Peer reviewed	Time period	1076
25	Nyberg *et al*^27^	UK	European	High income	2022	Peer reviewed	WGS	1 516 702
26	Paredes *et al*^32^	USA	Americas	High income	2022	Preprint+peer review	WGS	38 477
27	Pascall *et al*^37^	UK	European	High income	2022	Preprint	WGS	3854
28	Peralta-Santos *et al*^10^	Portugal	European	High income	2022	Preprint	WGS+SGTF	15 978
29	Raju *et al*^45^	India	Southeast Asian	Lower middle Income	2022	Peer reviewed	SGTF	1809
30	Robinson *et al*^25^	USA	Americas	High income	2022	Preprint+peer review	WGS	441
31	Sheikh *et al*^48^	Scotland	European	High income	2022	Peer reviewed	SGTF	143 504
32	Šmid *et al*^60^	Czech Republic	European	High income	2022	Peer reviewed	WGS+time period	No author response, hence excluded
33	Stålcrantz *et al*^35^	Norway	European	High income	2022	Peer reviewed	Unclear	1075
34	Wang *et al*^30^	USA	Americas	High income	2022	Preprint	Time period	294 214
35	Wang *et al*^54^	USA	Americas	High income	2022	Preprint	Time period	28 080
36	Wang *et al*^57^	USA	Americas	High income	2022	Preprint	Time period	14 396
37	Ward *et al*^31^	UK	European	High income	2022	Peer reviewed	SGTF	1 035 149
38	Whittaker *et al*^39^	Norway	European	High income	2022	Preprint+peer review	WGS+SGTF	125 269
39	Wolter *et al*^52^	South Africa	African	Upper middle income	2022	Peer reviewed	WGS+SGTF	11 495
40	Wrenn *et al*^44^	USA	Americas	High income	2022	Peer reviewed	WGS	752
Total								6 075 878
BA.1 versus BA.2								
1	Wolter *et al*^61^	South Africa	African	Upper middle income	2022	Preprint	SGTF	95 470
2	Gautret *et al*^62^	France	European	High income	2022	Peer reviewed	WGS	3000
3	Loconsole *et al*^63^	Italy	European	High income	2022	Peer reviewed	WGS+SGTF	459
Total								98 929

RdRP, RNA-dependent RNA P; RT-qPCR, quantitative reverse transcriptase PCR; SGTF, S-gene target failure; WGS, whole-genome sequencing.

For the Omicron versus Delta comparison, we were able to pool data from the studies for the following outcomes: death at longest follow-up, hospitalisation, ICU admission, receipt of oxygen therapy (low flow and high flow), and receipt of non-invasive and invasive ventilation.

**Figure 1:**
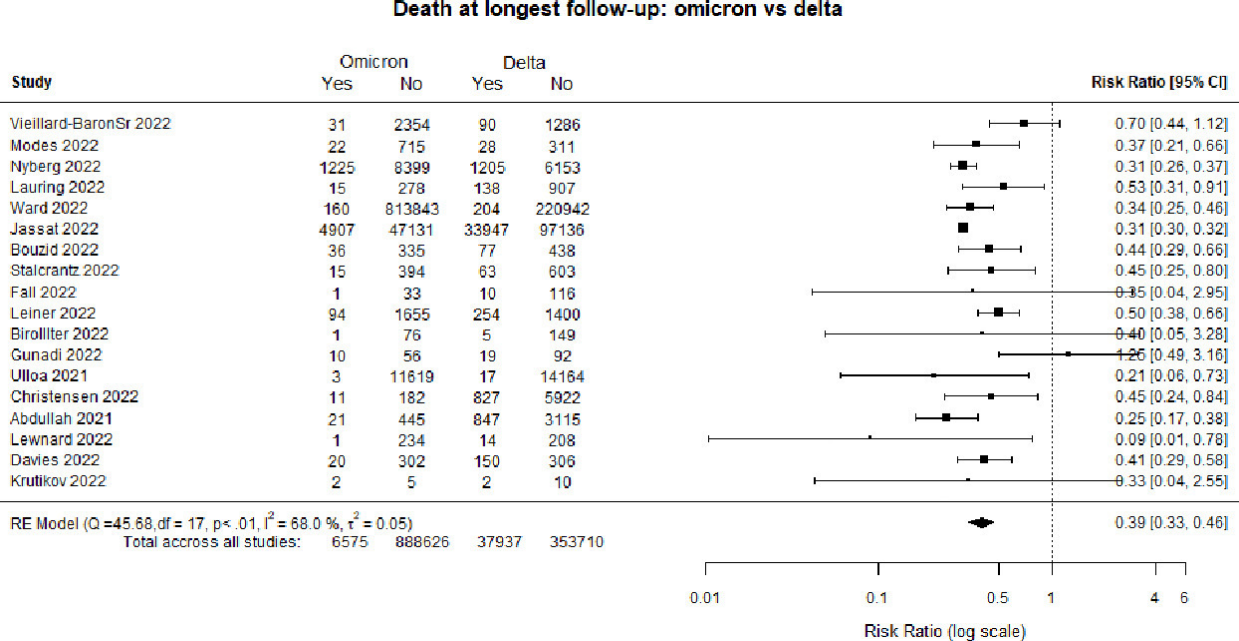
Forest plot of death: Omicron versus Delta. *Death at longest follow-up: studies included in the review followed patients for different time periods, some until hospital discharge, some for 14 days, some for 30 days etc. RE, Random Effects.

Figure 1 is a forest plot of death at the longest follow-up comparing patients infected with the Omicron as compared with Delta. Overall, individuals hospitalised with Omicron had a 61% lower risk of dying (RR 0.39, 95% CI :0.33 to 0.46).

**Figure 2:**
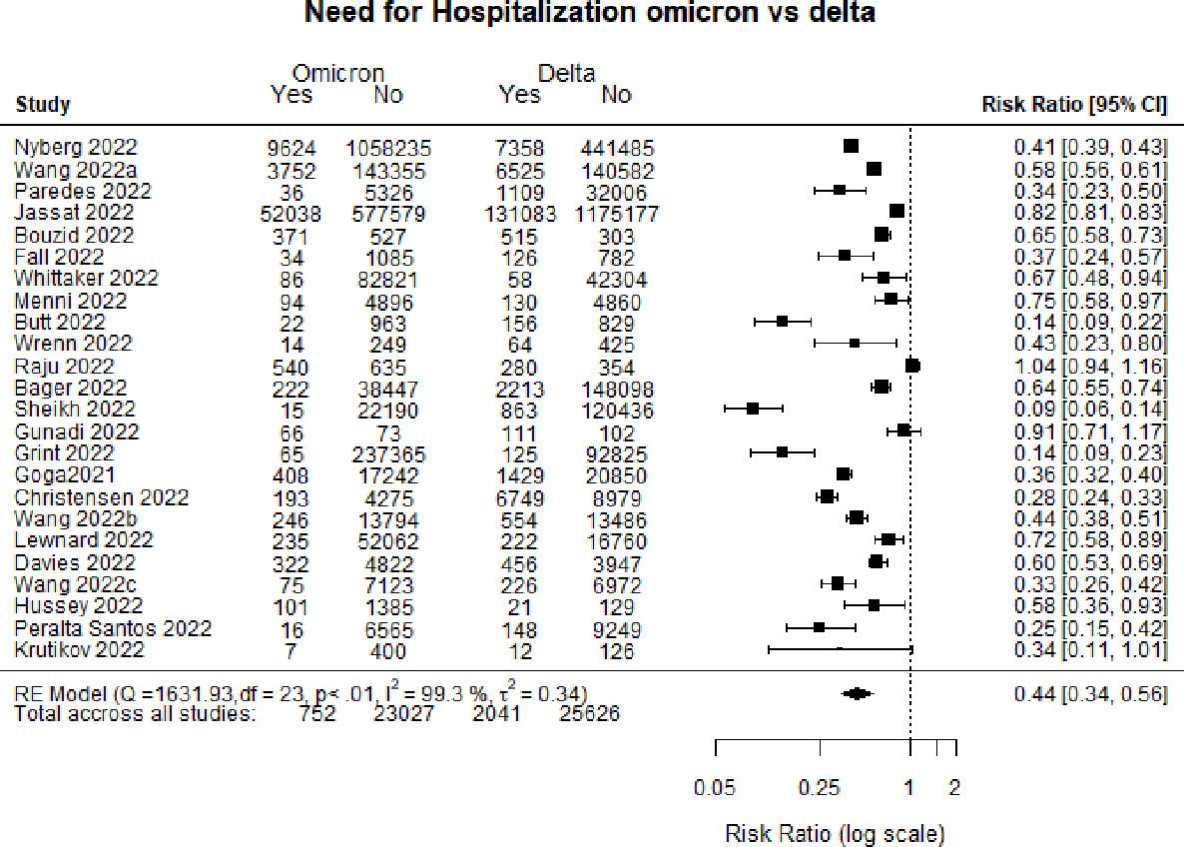
Forest plot comparing hospitalisation: Omicron versus Delta.

Figure 2 is a forest plot of hospitalisation comparing patients infected with the Omicron variant as compared with the Delta variant. Overall, individuals infected with Omicron had a 56% lower (RR 0.44, 95% CI 0.34 to 0.56) risk of being admitted to the hospital.

Similarly, patients infected with Omicron had a 54% lower risk (RR 0.46, 95% CI 0.37 to 0.57) of admission to ICU (online supplemental figure 1), 52% lower risk (RR 0.48, 95% CI 0.32 to 0.71) of receiving low-flow oxygen (online supplemental figure 2), 49% lower risk (RR 0.51, 95% CI 0.29 to 0.92) of receiving high-flow oxygen (online supplemental figure 3) and 59% lower risk (RR 0.41, 95% CI 0.29 to 0.57) of receiving invasive ventilation (online supplemental figure 4). There was no difference in the receipt of non-invasive ventilation between the variants (RR 0.90, 95% CI 0.69 to 1.19) (online supplemental figure 5).

**Figure 3:**
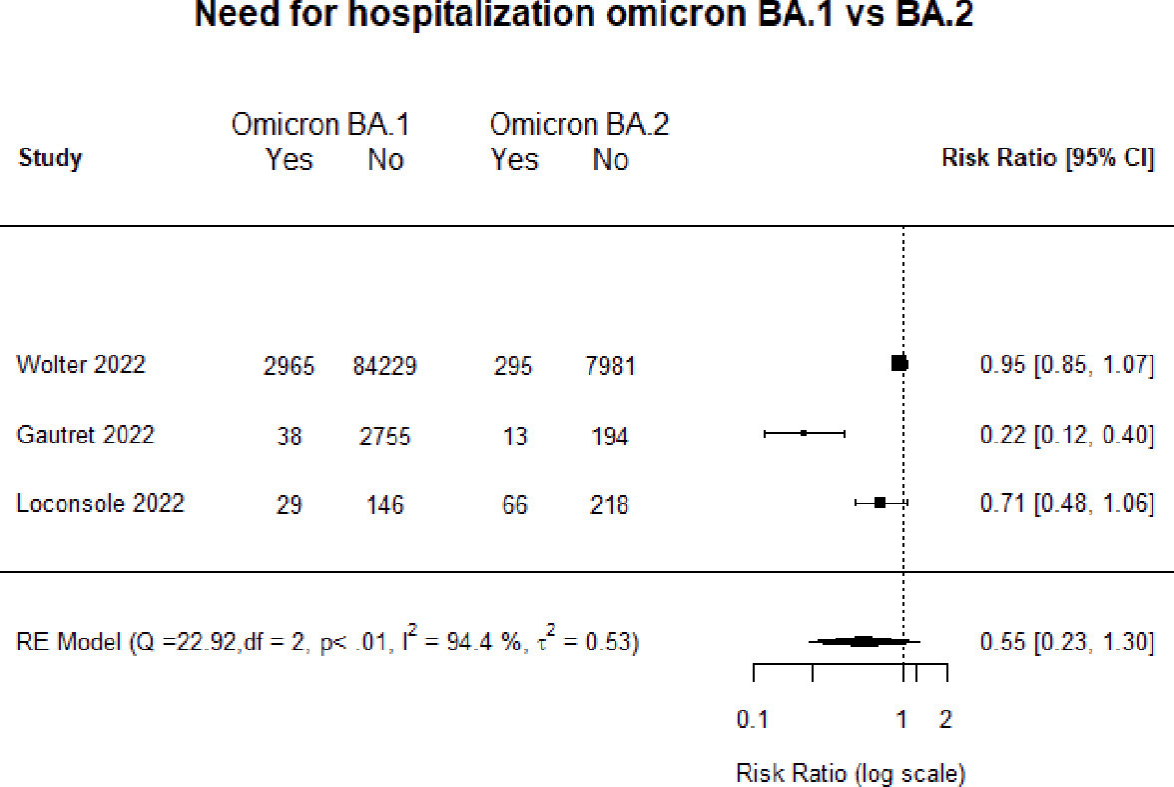
Forest plot comparing hospitalisation for Omicron sublineages.

For the BA.1 versus BA.2 comparison, we were able to pool studies only for the outcome of need for hospitalisation. Figure 3 is a forest plot comparing hospitalisation between the sublineages, and as can be seen, the risk of hospitalisation did not differ (RR 0.55, 95% CI 0.23 to 1.30).

### Sensitivity analysis

**Figure 4:**
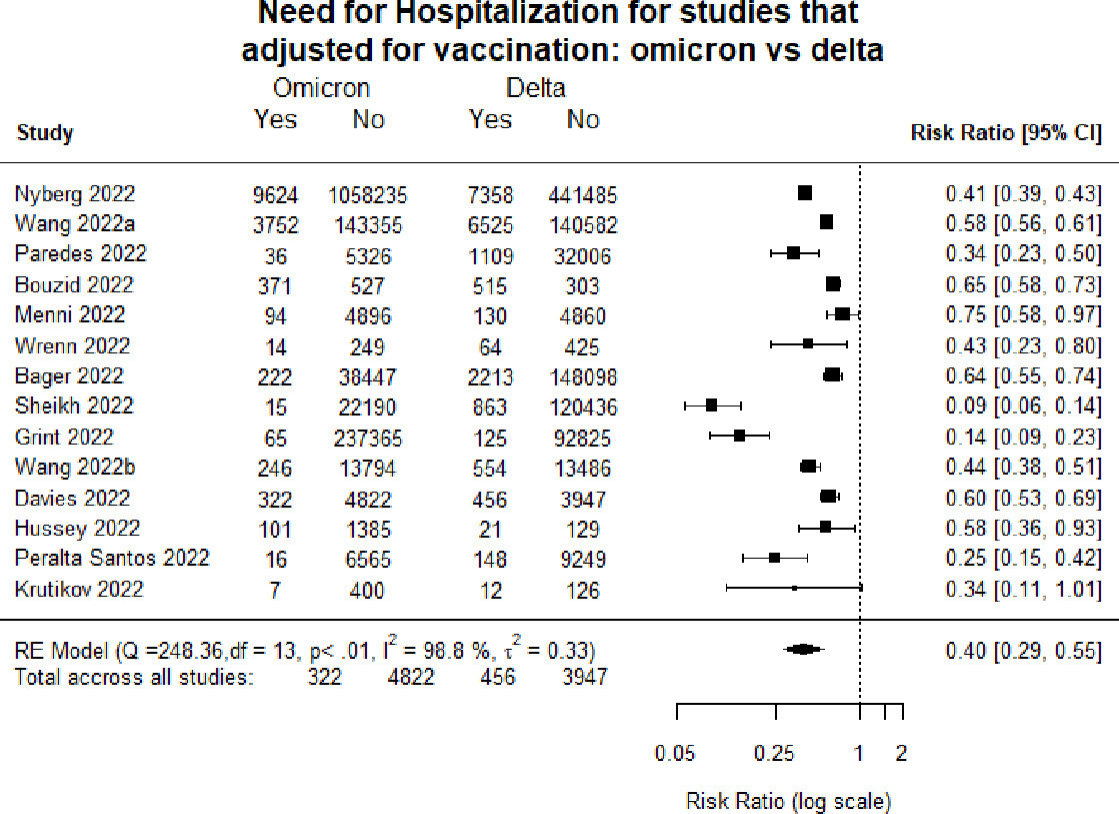
Forest plot comparing hospitalisation in studies adjusting for vaccination: Omicron versus Delta.

**Figure 5:**
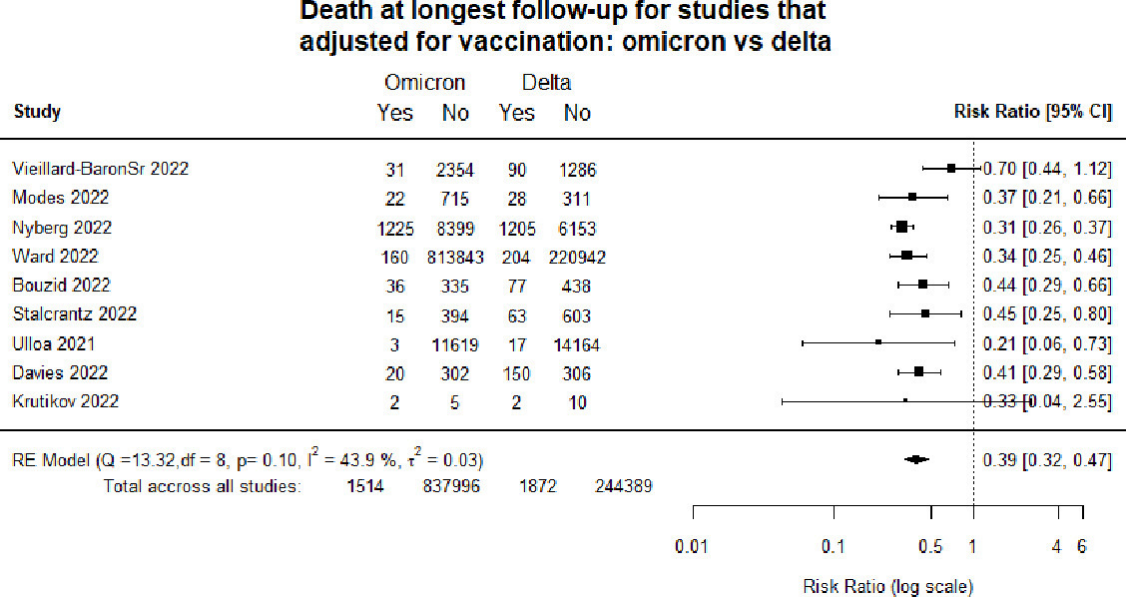
Forest plot comparing death in studies adjusting for vaccination: Omicron versus Delta.

Figures 4 and 5 show hospitalisation and death when pooling only studies that explicitly adjusted for vaccination. This analysis found similar results regarding the lower risk of hospitalisation and death following infection with Omicron compared with infection with Delta.

Similarly, pooling only studies with a low RoB (online supplemental figure 6 and 7) found that the risk of hospitalisation and death was lower with infection with Omicron compared with infection with Delta.

### Study quality

Online supplemental figure 8 provides a visual representation of the quality of the included studies. Nearly half of the studies were at high RoB.

## Discussion

Our systematic review comparing outcomes for the Omicron variant as compared with Delta shows a lower risk of death and hospitalisation. There was also a lower risk of receipt of oxygen therapy, and invasive ventilation. Our sensitivity analysis pooling only studies that explicitly adjusted for vaccination and studies at low RoB also corroborates these findings. Comparing sublineages, the results show that the risk of hospitalisation did not differ between BA.1 versus BA.2.

These results broadly corroborate those of an earlier WHO report^12^ which suggested lower odds of developing severe or critical disease (OR 0.43, 95% CI 0.41 to 0.46) and lower hazards of in-hospital mortality (HR 0.62, 95% CI 0.59 to 0.65), but add more confidence and precision to the estimates as our study included over 300 times the number of patients and covers more countries than those included in the WHO report. Prior studies have demonstrated enhanced transmissibility with Omicron^64^ and the capacity for immune evasion.^64 65^ Considering these characteristics that confer a ‘fitness advantage’, we found that our data demonstrating lower clinical severity is reassuring.

There are various factors that may explain the lower observed severity and mortality of Omicron compared with Delta. Higher levels of both infection-derived and vaccine-derived immunity may confound the association, resulting in lower apparent severity of infection particularly as immunity from vaccines and infection protects against severity and hospitalisation more than it protects against infection.^66 67^ Although the analysis limited to studies adjusting for vaccination showed a reduced severity from Omicron, there was inadequate adjustment for immunity from prior infection, which is likely to partially lower the overall risk too. Also, our analysis showed no statistical difference in severity between BA.1 and BA.2, and other studies comparing multiple waves of Omicron have not observed substantial changes in severity for more recent variants (BA.4/BA.5 compared with BA.1/BA.2).^68^

In our review, we have considered severity and outcomes from two perspectives, hospitalisation among infected and in-hospital outcomes for those that needed admission.^69^ However, it must be noted that hospitalisation as a measure of severity is not very specific; it can often ‘be with COVID-19’ rather than ‘because of COVID-19’. Despite this limitation, we included hospitalisation as a key outcome as it represents a concrete measure of healthcare use and is also a measure of the burden on potentially overwhelmed healthcare systems.

As such, while higher population immunity from prior infection and vaccination during Omicron waves of 2022 and 2023 may explain some of the reduced severity observed, it is likely that the intrinsic severity of Omicron is lower than that of previous variants, potentially due to its tropism for upper airways and hence lower risk of lower respiratory tract infection, as suggested by in vitro studies.^70 71^

Assessing the impact of new and emerging variants on severity and clinical outcomes remains critical as the pandemic continues. With waning immunity, changes in testing, surveillance and reporting, cocirculation of multiple lineages and variability in clinical management, assessing severity for each new emerging variant is essential in order to optimise clinical care and reduce the impact of COVID-19. COVID-19 surveillance must continue to be strengthened globally and include linking epidemiological, laboratory and clinical data. It is also important to note that most studies in our review come from high-income settings. There is thus a need to understand outcomes from newer variants and sublineages from low-income and lower middle-income settings.

### Strengths and limitations

This is the first systematic review comparing clinical severity and outcomes for individuals infected with Omicron as compared with Delta. Our results provide important information for healthcare providers, public health officials, ministries of health and other policy makers around the world. Our review followed rigorous methods: preregistration of the protocol, adherence to MOOSE and PRISMA guidelines with duplicate and independent screening, data extraction and quality assessment.

Our review also has some limitations. First, nearly half of the included studies were at high RoB. About a third of included studies did not adjust for vaccination status, and this could have impacted the results. However, this is mitigated somewhat by the consistency of our results across the several outcomes and results from our sensitivity analysis. In recent months, several newer sublineages of Omicron have emerged. While our search strategy included BA.4 and BA.5, there were no published studies on these variants at the time of our search. Thus, our review does not provide information on these sublineages of Omicron, including those that are driving a 2023 surge in China (BF.7).^72^ We were unable to compare outcomes across different age groups and also unable to perform a sex-disaggregated analysis. Additional limitations of the results of the review include the substantial heterogeneity between studies and the fact that a quarter of the studies were only published as preprints, thus potentially limiting the generalisability of the results.

## Conclusion

The Omicron variant as compared with Delta was associated with a lower risk of death and hospitalisation. There was also a lower risk of receipt of oxygen therapy, non-invasive ventilation and invasive ventilation; however, disentangling the relative effect of immunity from variant-specific properties remains challenging. Comparing sublineages, this study shows that the risk of hospitalisation did not differ between BA.1 versus BA.2.

## Data Availability

All data relevant to the study are included in the article or uploaded as supplementary information.
