# Supplementary material for: Severity and outcomes of Omicron variant of SARS-CoV-2 compared to Delta variant and severity of Omicron sublineages: a systematic review and metanalysis
Source: BMJ Glob Health. 2023 Jul 7;8(7):e012328. doi: 10.1136/bmjgh-2023-012328 (PMC10347449; doi:10.1136/bmjgh-2023-012328)
Supplement: Supplementary file 1 [file bmjgh-2023-012328supp001.pdf]

SUPPLEMENTARY MATERIAL

Title: Severity and outcomes of Omicron variant of SARS-CoV2 as compared to the Delta variant and severity of Omicron sublineages: A systematic review and metanalysis

Authors: Pryanka Relan , Nkengafac Villyen Motaze, Kavita U. Kothari, Lisa Askie, Olivier le Polain de Waroux, Maria D. Van Kerkhove, Janet Diaz, Bharath Kumar Tirupakuzhi Vijayaraghavan

Contents

1. *PECO style presentation of systematic review* 2
2. *Details of Search strategy* 3
3. *WHO COVID-19 Research Database Sources* 5
4. *Additional outcomes* 6

Suppl. Figure 1: Forest plot comparing ICU admission – Omicron vs Delta 6

Suppl. Figure 2: Forest plot comparing Oxygen therapy – Omicron vs Delta 7

Suppl. Figure 3: Forest plot comparing high flow nasal cannula – Omicron vs Delta 8

Suppl. Figure 4: Forest plot comparing invasive ventilation – Omicron vs Delta 9

Suppl. Figure 5: Forest plot comparing receipt of non-invasive ventilation-Omicron vs. Delta 10

Suppl. Figure 6: Forest plot comparing need for hospitalization including only studies at low risk of bias – Omicron vs Delta 11

Suppl. Figure 7: Forest plot comparing death at longest follow-up including only studies at low risk of bias – Omicron vs Delta 12

Suppl. Figure 8: Risk of Bias in included studies 13

1. PECO style presentation of systematic review

Population:

All patients (adults and children) who tested positive for SARS-CoV-2 (using any standard diagnostic test) and were infected with Delta or Omicron. For the BA.1 vs. BA.2 comparison, population was similarly defined.

Exposure:

Infection with Omicron variant identified as per study investigators (could be based on Whole Genome Sequencing (WGS), S-gene Target Failure (SGTF), time-periods or any other method)

For the BA.1 vs BA.2 comparison, exposure was defined as infection with BA.2 sublineage as identified by study investigators.

Comparator:

Infection with the Delta variant identified as per study investigators and BA.1 for the sublineage comparison.

Outcomes:

For both review questions, severity was assessed by comparing hospitalization, intensive care unit admission, receipt of oxygen therapy (low or high-flow), receipt of non-invasive or invasive ventilation, receipt of vasopressors, receipt of kidney replacement therapy and receipt of any other organ support (e.g. ExtraCorporeal Membrane Oyxgenation or ECMO). Clinical outcomes were assessed by comparing survival, length of hospital stay and post discharge outcomes such as quality of life, if reported.

2. Details of Search strategy

First search run 26 January 2022.

Concept	Search string	Results
#1- VOC	*Omicron* OR *B.1.1.529* OR *B*1*1*529* OR "Ba.1" OR "Ba.2" OR "ba.3" OR "BA.11" OR "VUI 22 JAN 01"	703
#3 - Prognosis -	(Validat* OR Predict* OR prognos* OR history* OR course OR "follow-up" OR Cohort* OR longitudinal OR prospective OR history OR surviv* OR outcome OR severity OR observa* OR Scor* OR clinical* OR Risk* OR odds)	220.408
#4	#1 AND #3<br><br>((*Omicron* OR *B.1.1.529* OR *B*1*1*529* OR "Ba.1" OR "Ba.2" OR "ba.3" OR "BA.11" OR "VUI 22 JAN 01") AND (Validat* OR Predict* OR prognos* OR history* OR course OR "follow-up" OR Cohort* OR longitudinal OR prospective OR history OR surviv* OR outcome OR severity OR observa* OR Scor* OR clinical* OR Risk* OR odds))	309

Second search run on 16 May 2022. There were 1185 citations since 26-01-2022 till 16-05-2022.

Concept	Search string	Results
#1- VOC	*Omicron* OR *B.1.1.529* OR *B*1*1*529* OR "Ba.1" OR "Ba.2" OR "ba.3" OR "BA.11" OR "VOC 21NOV 01" OR "VUI 22 JAN 01" OR "BA.1.1" OR "BA.4" OR "BA.5" OR "BA.2.12.1" OR "BA.2.9.1" OR "BA.2.11" OR "BA.2.13" OR "variant 21K" OR "Variant 21L" OR subvariant* OR "sub variant" OR "sub variants"	3175
#3 - Prognosis -	(Validat* OR Predict* OR prognos* OR history* OR course OR "follow-up" OR Cohort* OR longitudinal OR prospective OR history OR surviv* OR outcome OR severity OR observa* OR Scor* OR clinical* OR Risk* OR odds)	288,283
#4	#1 AND #3<br><br>((*Omicron* OR *B.1.1.529* OR *B*1*1*529* OR "Ba.1" OR "Ba.2" OR "ba.3" OR "BA.11" OR "VOC 21NOV 01" OR "VUI 22 JAN 01" OR "BA.1.1" OR "BA.4" OR "BA.5" OR "BA.2.12.1" OR "BA.2.9.1" OR "BA.2.11" OR "BA.2.13" OR "variant 21K" OR "Variant 21L" OR subvariant* OR "sub variant" OR "sub variants") AND (Validat* OR Predict* OR prognos* OR history* OR course OR "follow-up" OR Cohort* OR longitudinal OR prospective OR history OR surviv* OR outcome OR severity OR observa* OR Scor* OR clinical* OR Risk* OR odds))	1509
#5	entry_date:([20220126 TO 20220516])	119.925
#6	#4 AND #5<br><br>((*Omicron* OR *B.1.1.529* OR *B*1*1*529* OR "Ba.1" OR "Ba.2" OR "ba.3" OR "BA.11" OR "VOC 21NOV 01" OR "VUI 22 JAN 01" OR "BA.1.1" OR "BA.4" OR "BA.5" OR "BA.2.12.1" OR "BA.2.9.1" OR "BA.2.11" OR "BA.2.13" OR "variant 21K" OR "Variant 21L" OR subvariant* OR "sub variant" OR "sub variants") AND (Validat* OR Predict* OR prognos* OR history* OR course OR "follow-up" OR Cohort* OR longitudinal OR prospective OR history OR surviv* OR outcome OR severity OR observa* OR Scor* OR clinical* OR Risk* OR odds)) AND (entry_date:([20220126 TO 20220516]))	**1185**

3. WHO COVID-19 Research Database Sources

- MedRxiv
- BioRxiv
- ICTRP
- Medline (Ovid)
- Medline (PubMed)
- CAB Abstracts (Ovid)
- Global Health (Ovid)
- PsycInfo (Ovid)
- Scopus (Elsevier)
- Academic Search Complete (Ebsco)
- Africa Wide Information (Ebsco)
- CINAHL (Ebsco)
- ProQuest Central (ProQuest)
- Agricultural & Environmental Science Collection, Coronavirus Research Database, ERIC, ProQuest Central, PTSDpubs, Sociological Abstracts
- EuropePMC
- PubMed
- PubMed Central
- Agricola
- 21 preprint servers including AAS Open Research, AMRC Open Research, arXiv, Authorea, Beilstein, BioHackrXiv, bioRxiv, chemRxiv, Emerald Open Research, F1000, Gates Open Research, HRB Open Research, medRxiv, MNI Open Research, Open Research Europe, PeerJ, Preprints.org, psyArXiv, Research Square, SSRN, and Wellcome Open Research
- PubMed Central
- China CDC MMWR
- CDC Guidelines (US CDC)
- chemRxiv
- SSRN
- Embase (OVID)
- National Bureau of Economic Research
- Social Science Open Access Repository
- Web of Science
- Science Direct
- Taylor and Francis
- MDPI
- IRIS

4. *Additional outcomes*

Suppl. Figure 1: Forest plot comparing ICU admission – Omicron vs Delta

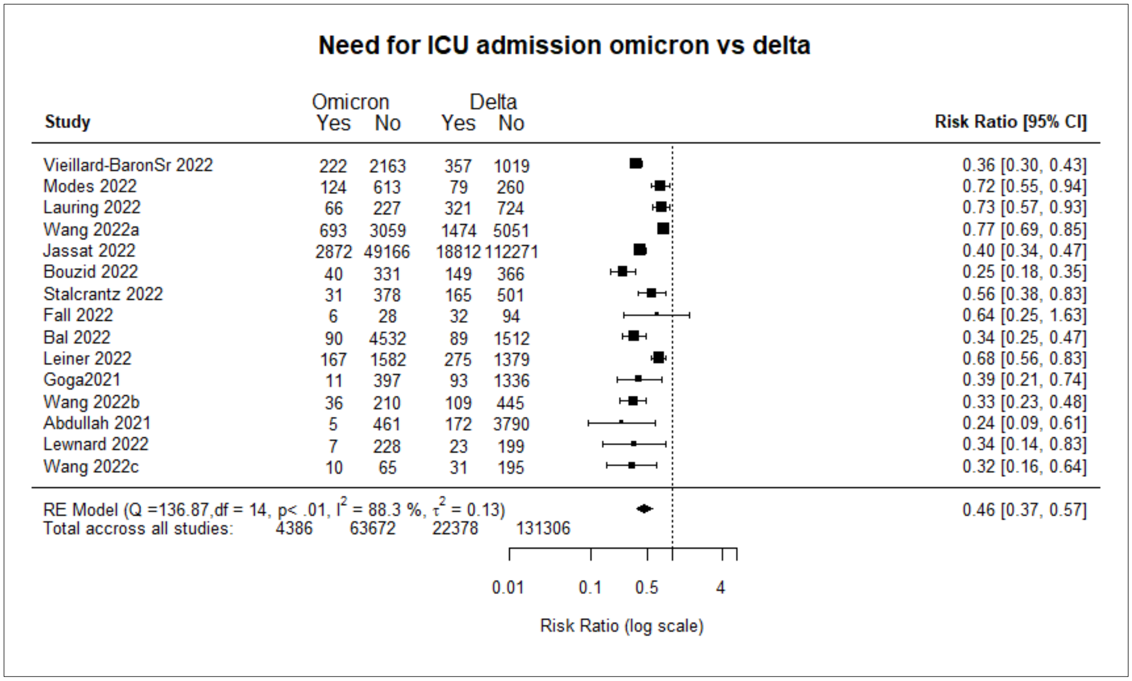

Suppl. Figure 2: Forest plot comparing Oxygen therapy – Omicron vs Delta

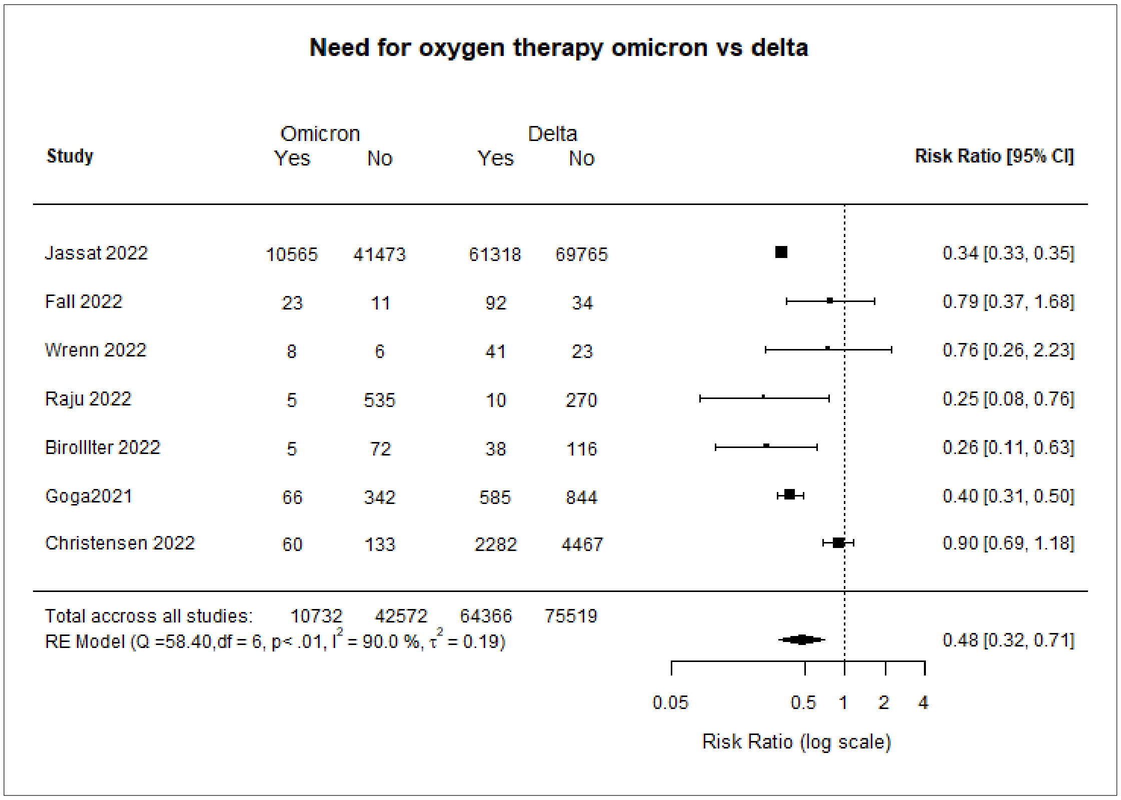

Suppl. Figure 3: Forest plot comparing high flow nasal cannula – Omicron vs Delta

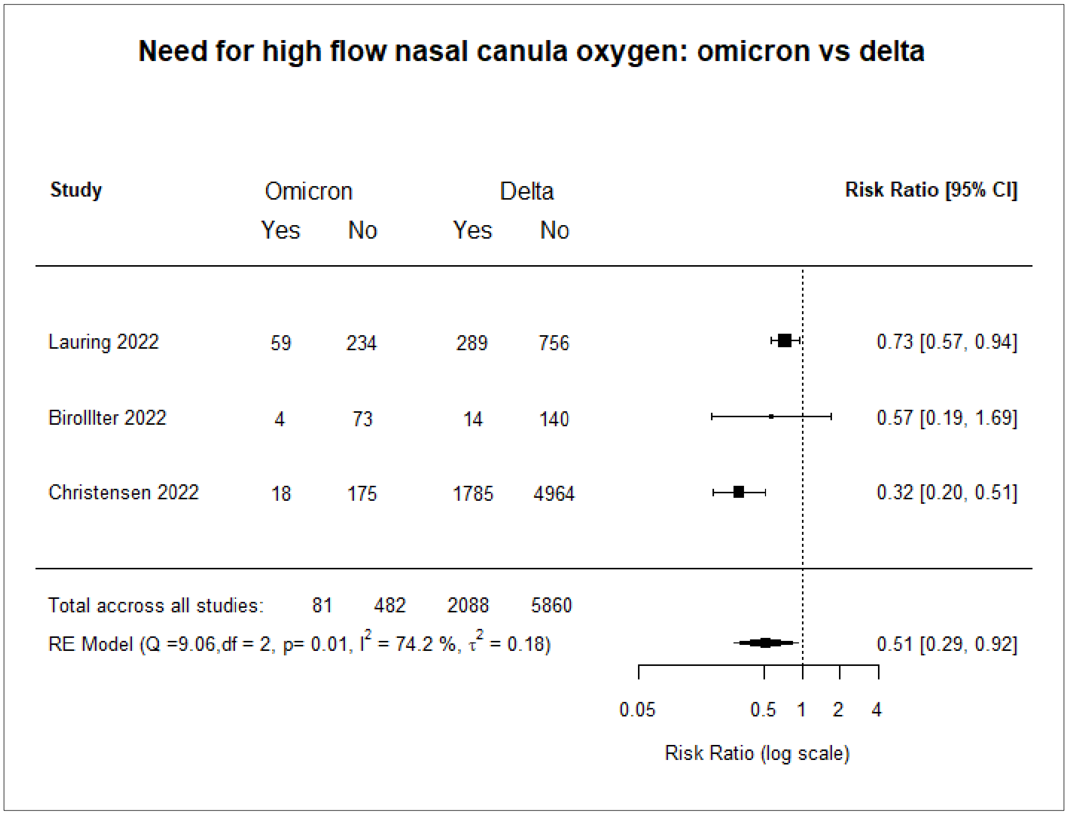

Suppl. Figure 4: Forest plot comparing invasive ventilation – Omicron vs Delta

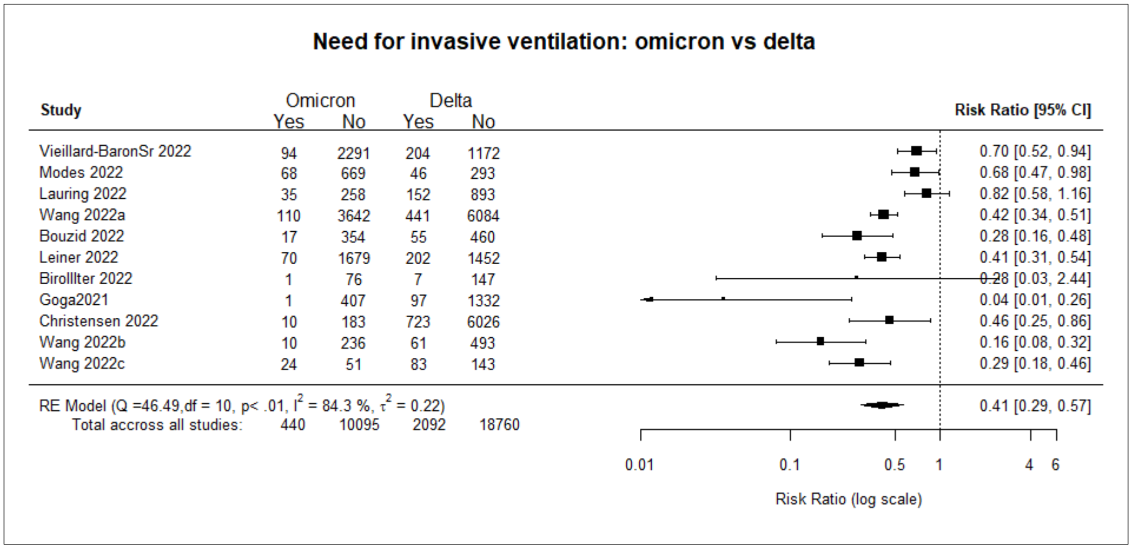

Suppl. Figure 5: Forest plot comparing receipt of non-invasive ventilation-Omicron vs. Delta

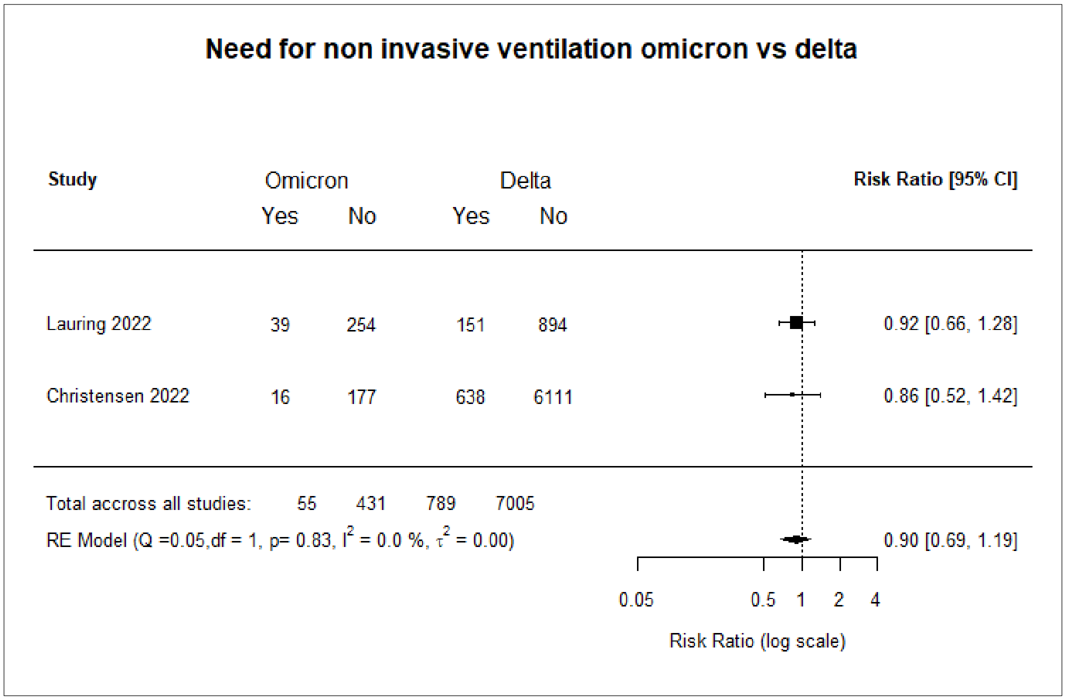

Suppl. Figure 6: Forest plot comparing need for hospitalization including only studies at low risk of bias – Omicron vs Delta

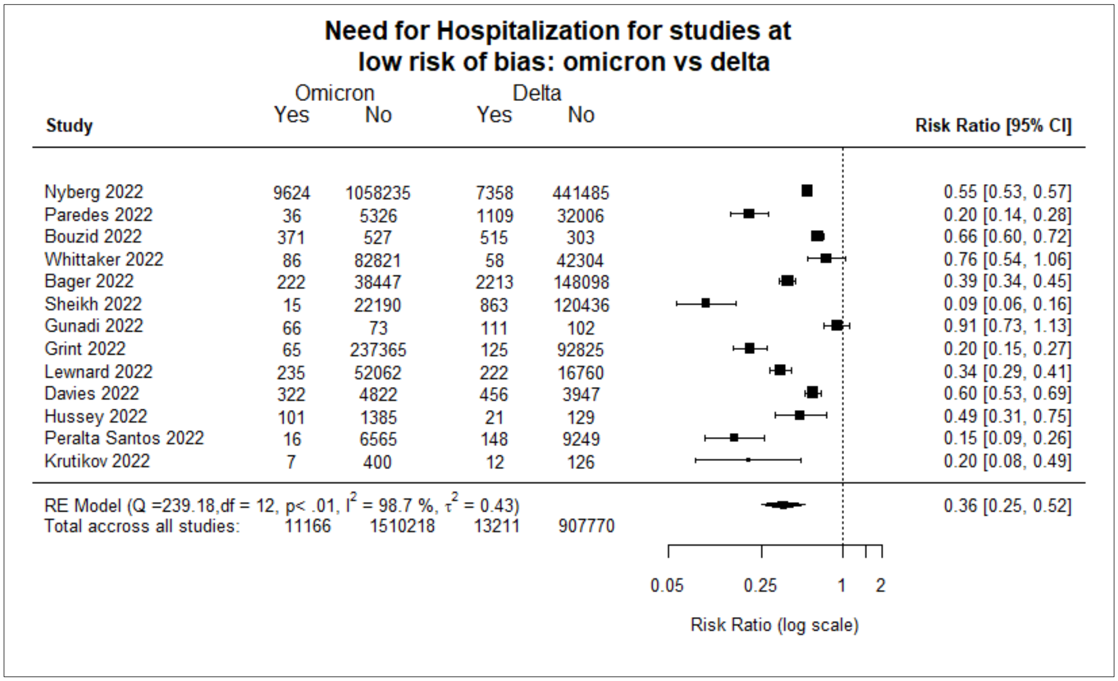

Suppl. Figure 7: Forest plot comparing death at longest follow-up including only studies at low risk of bias – Omicron vs Delta

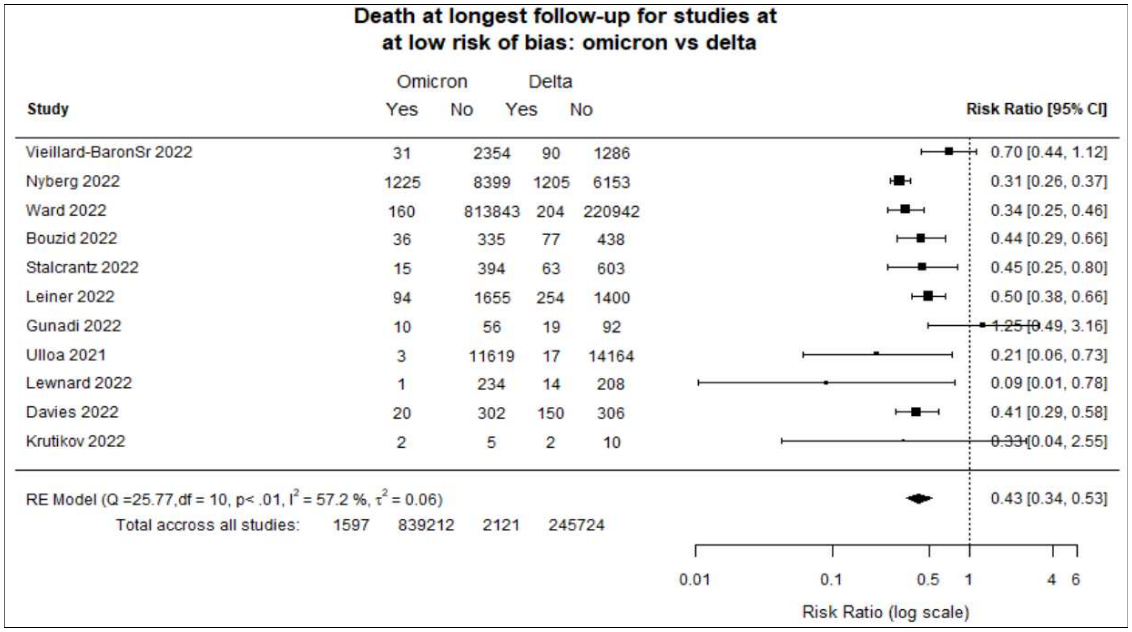

Relan P, *et al. BMJ Glob Health* 2023; 8:e012328. doi: 10.1136/bmjgh-2023-012328

Suppl. Figure 8: Risk of Bias in included studies

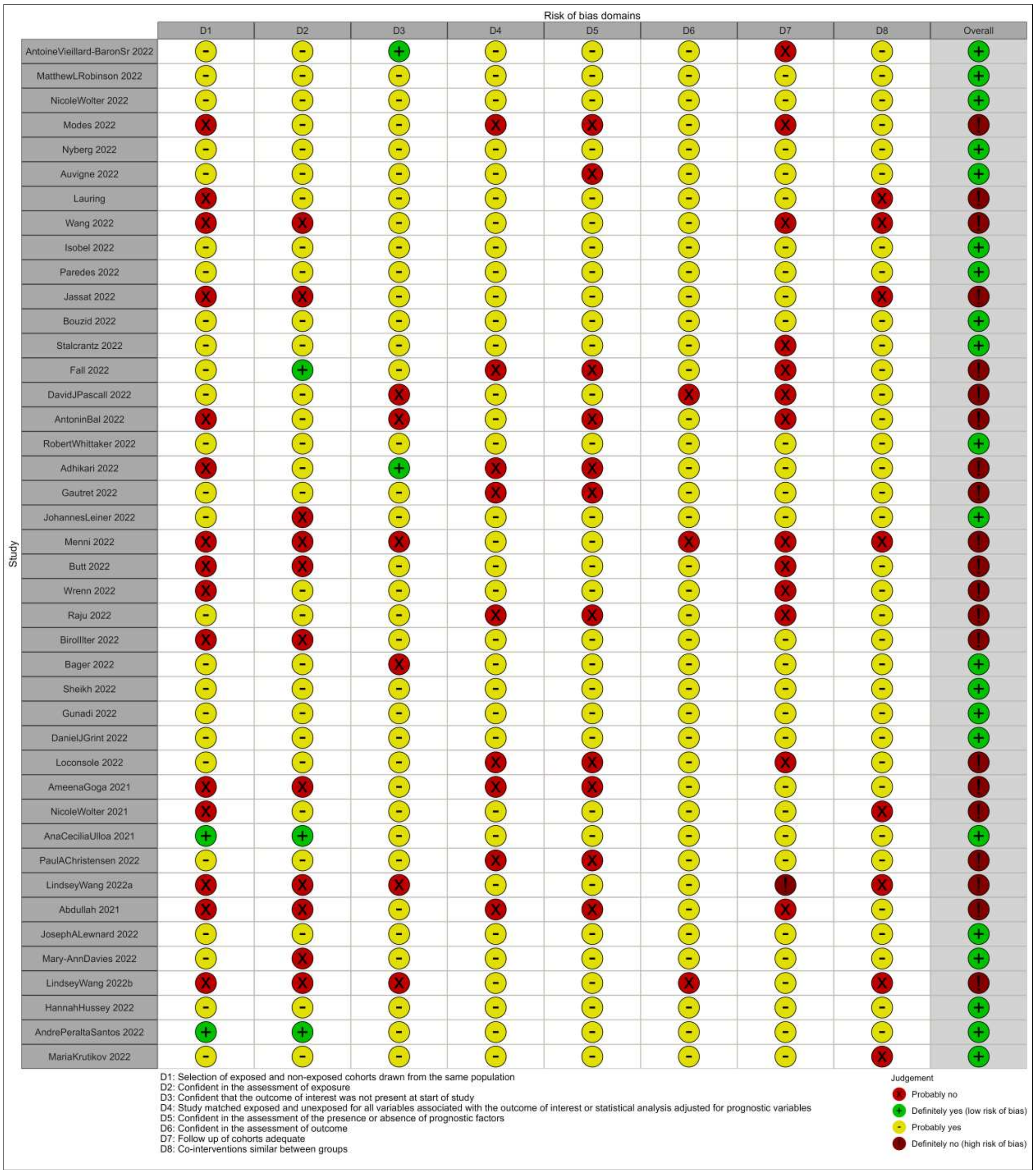

***For overall study rating, we used apriori defined empiric rules. If a study had a 'definite no' for any of the domains and a 'probably no' for 2 or more domains, the study was classified as being at 'high risk of bias'**
